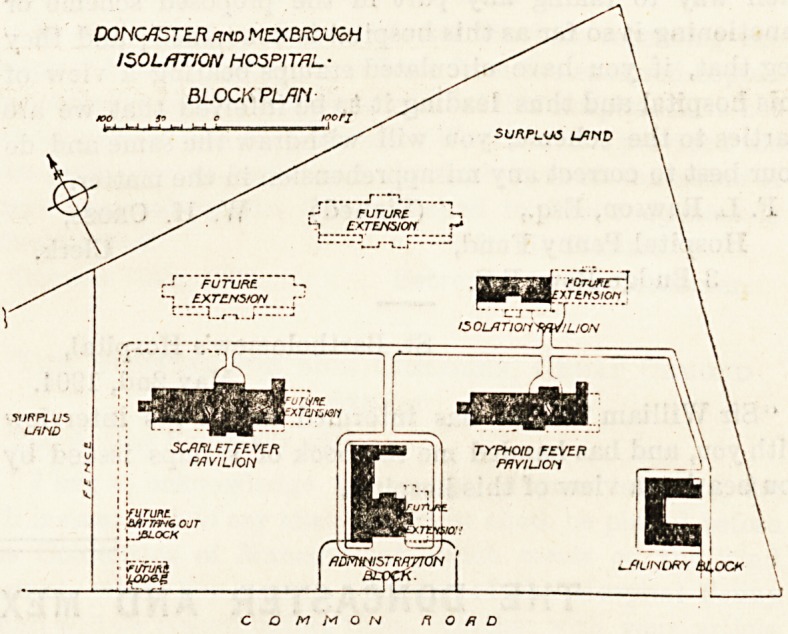# The Doncaster and Mexboro' Infectious Hospital

**Published:** 1904-10-15

**Authors:** 


### THE DONCASTER AND MEXBORO' INFECTIOUS HOSPITAL.

This hospital has been built at Conisbro' on a site of 7⅓ acres in extent, and about three-quarters of a mile from the railway station. It was opened in February 1904. It consists of five separate blocks—namely, the administrative, the laundry, the scarlet fever, the typhoid, and the isolation pavilion. In shape the plot of land is triangular, and the administrative block is placed near the south boundary and overlooks the common road. The entrance hall has on one side the medical officers' sitting-room, and on the other the matron's room, the former being reached direct from the hall, the latter from a corridor which runs east and west through the block. At the west end is the nurses' sitting-room, and near it are the nurses' dining-room, the servants' hall, staircase, and linen-room. Further north is the kitchen department, which is conveniently arranged, and partly encloses the kitchen yard. The first floor of this block contains nine bedrooms and a bath-room, and the second floor has four bedrooms and a box-room.

The laundry department occupies the south east angle of the plot, and it consists of washhouse, ironing-room, drying-rooms, and it has incorporated with it the boiler-house, disinfecting-rooms, and electric light installation plant.

The scarlet-fever pavilion is on the north-west side of the administrative department. It faces south, and both of its large wards are provided with verandahs. The accommodation consists of a ten-bedded ward, a six-bedded ward, and two single-bedded rooms, the latter being intended for paying patients. These special or private patients' wards project several feet to the south of the large wards, and each has a window in the south elevation and another window opens into the verandah. By this means some cross-ventilation will be obtainable, but it is not equal to the direct current between windows placed opposite each other. The closets and sinks are placed at the ends of the large wards and are cut off from the wards by cross-ventilated lobbies. The nurses' duty-room is placed between the wards and is provided with inspection windows. The bath-rooms are situated on the north side, and form a sort of balance to the special wards on the south. There is a small store-room and a

The architect was Mr. J. H. Morton, of South Shields; the contractors were Messrs. Arnold and Son, of Doncaster. Messrs. Benham and Son, of London, held the sub-contract linen-room, and a staircase leads to a good-sized day-room, this being in the centre of the block, and is the only part carried up to a second story.

The typhoid block has similar accommodation, and it is arranged in a line with the scarlet-fever pavilion, and is further east. To the north of the typhoid block is that for the isolation of doubtful cases, and for other varieties of infectious diseases. It is smaller than the others.

The board wisely bought more land than they required to begin with, and therefore there is room for future extension by at least two additional pavilions. Accommodation is already provided for 36 patients, and this should meet the requirements of the united district for a considerable time to come. It is probable, however, that the board may find it desirable te erect a lodge, a discharging block, and a mortuary before long.

The engineering work has been carried out on the most approved and modern lines; and it is not likely that either it or the administration part would need enlarging when it becomes necessary to build other pavilions. The hospital has been appropriately furnished throughout; and telephonic communication exists between the various blocks.

The cost of the whole hospital in its present state works out at £339 per bed; and of course this cost per bed would be very considerably reduced if pavilions were to be erected for 30 additional cases.

for the engineering work, and Mr. Frederick Simpson was the clerk of the works.

## Figures and Tables

**Figure f1:**
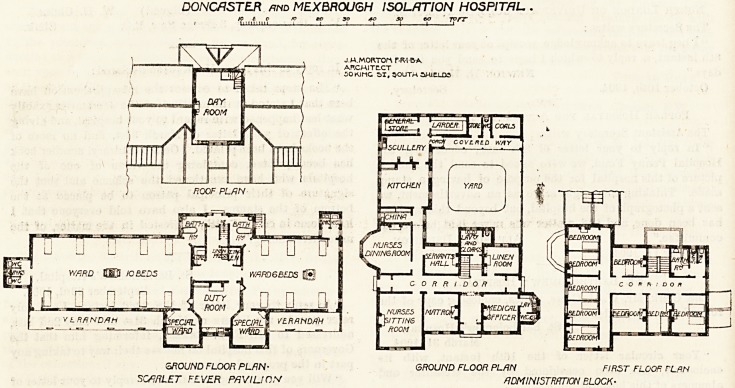


**Figure f2:**
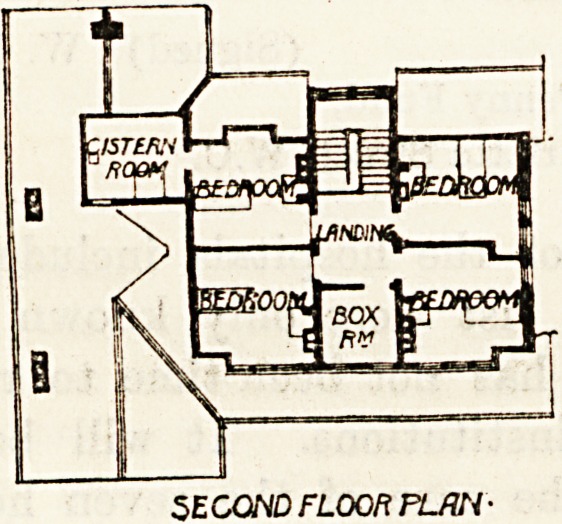


**Figure f3:**